# Coronavirus Papain-like Proteases Negatively Regulate Antiviral Innate Immune Response through Disruption of STING-Mediated Signaling

**DOI:** 10.1371/journal.pone.0030802

**Published:** 2012-02-01

**Authors:** Li Sun, Yaling Xing, Xiaojuan Chen, Yang Zheng, Yudong Yang, Daniel B. Nichols, Mark A. Clementz, Bridget S. Banach, Kui Li, Susan C. Baker, Zhongbin Chen

**Affiliations:** 1 Division of Infection and Immunity, Department of Electromagnetic and Laser Biology, Beijing Institute of Radiation Medicine, Beijing, China; 2 Department of Microbiology and Immunology, Loyola University of Chicago Stritch School of Medicine, Maywood, Illinois, United States of America; 3 Department of Microbiology, Immunology and Biochemistry, University of Tennessee Health Science Center, Memphis, Tennessee, United States of America; McMaster University, Canada

## Abstract

Viruses have evolved elaborate mechanisms to evade or inactivate the complex system of sensors and signaling molecules that make up the host innate immune response. Here we show that human coronavirus (HCoV) NL63 and severe acute respiratory syndrome (SARS) CoV papain-like proteases (PLP) antagonize innate immune signaling mediated by STING (stimulator of interferon genes, also known as MITA/ERIS/MYPS). STING resides in the endoplasmic reticulum and upon activation, forms dimers which assemble with MAVS, TBK-1 and IKKε, leading to IRF-3 activation and subsequent induction of interferon (IFN). We found that expression of the membrane anchored PLP domain from human HCoV-NL63 (PLP2-TM) or SARS-CoV (PLpro-TM) inhibits STING-mediated activation of IRF-3 nuclear translocation and induction of IRF-3 dependent promoters. Both catalytically active and inactive forms of CoV PLPs co-immunoprecipitated with STING, and viral replicase proteins co-localize with STING in HCoV-NL63-infected cells. Ectopic expression of catalytically active PLP2-TM blocks STING dimer formation and negatively regulates assembly of STING-MAVS-TBK1/IKKε complexes required for activation of IRF-3. STING dimerization was also substantially reduced in cells infected with SARS-CoV. Furthermore, the level of ubiquitinated forms of STING, RIG-I, TBK1 and IRF-3 are reduced in cells expressing wild type or catalytic mutants of PLP2-TM, likely contributing to disruption of signaling required for IFN induction. These results describe a new mechanism used by CoVs in which CoV PLPs negatively regulate antiviral defenses by disrupting the STING-mediated IFN induction.

## Introduction

The innate immune system is the first line of defense that protects the host against viral infection. Viral infections are sensed by pattern-recognition receptors (PRRs) of the innate immune system that recognize pathogen-associated molecular patterns (PAMPs) and then trigger an antiviral response [1]. Viral nucleic acids, such as the viral genome or replicative intermediates produced during viral replication, can be recognized by toll-like receptors (TLR3/7/8/9) or the retinoid acid-inducible gene (RIG)-I-like helicase (RLH) family members RIG-I and melanoma differentiation-associated protein 5 (MDA-5) [2], [3]. Viral double stranded RNA can be sensed by membrane bound TLRs or cytosolic sensors like MDA-5, whereas RIG-I detects intracellular viral RNAs bearing 5′-triphosphate ends with base-paired structures to activate antiviral signaling [4]–[7]. Upon engagement with viral RNA, these PRRs recruit different adaptor proteins (MAVS/IPS-1/VISA/Cardif for RIG-I, and TRIF for TLR3 and MyD88 for TLR7/8/9), and transduce signals to the downstream kinase complexes which activate IFN regulatory factor-3 (IRF-3), nuclear factor κB (NF-κB) and ATF-2/c-jun. These transcription factors coordinately regulate the expression of type I Interferons (IFN-β and -α). Type I IFNs induce the activation of STAT transcription factors that induce the expression of hundreds of IFN-stimulated genes (ISGs) which establish an antiviral state in surrounding cells, thereby limiting viral replication and spread.

Recent investigations into the induction of the type I IFN response identified a new player in the pathway, designated here as STING (stimulator of interferon genes; also called MITA, ERIS and MPYS) [8]–[11]. STING was identified by investigators screening cDNA libraries for genes that, when overexpressed, were sufficient to activate production of IFN. Further studies revealed that STING-knockout mice are susceptible to lethal infection with herpes simplex virus 1 and vesicular stomatitis virus, demonstrating the critical role of STING in facilitating immune responses to viral pathogens [12]. STING, with four transmembrane domains in the N-terminal region, is detected in the endoplasmic reticulum (ER) and upon activation complexes with signaling components including TBK1, leading to phosphorylation of IRF-3 [8]. In addition, activation of STING induces its dimerization and ubiquitination, which are proposed to play important roles in the activation of IRF-3 signaling [9].

**Figure 1:**
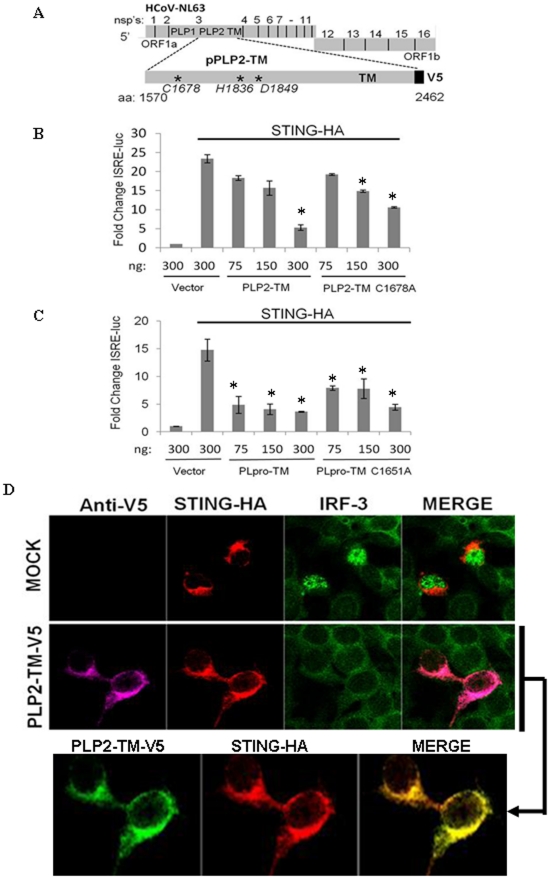
Expression of coronavirus PLPs blocks STING-mediated activation of the interferon stimulated response element (ISRE). (**A**) Schematic diagram of human coronaviruses (HCoV) NL63 illustrating the processing of replicase polyproteins to generate nonstructural proteins (nsp's). The papain-like protease domains, the catalytic residues that essential for protease catalytic activity [35], and the transmembrane (TM) domain within nsp3 are indicated. (**B** and **C**) HEK293T cells were transfected with the STING-HA, ISRE-luc reporter and either wild-type or catalytic mutants of HCoV-NL63 PLP2-TM or SARS-CoV PLpro-TM. Asterisks indicate statistical significance (P<0.05) in comparison with ISRE-reporter activity stimulated with STING. (**D**) Immunofluorescence microscopy of HEK-293T cells expressing STING-HA and PLP2-TM-V5. Cells were fixed at 24 hrs post-transfection and the localization of endogenous IRF-3 (anti-IRF-3, green) and the epitope-tagged products was visualized by confocal microscopy.

Coronaviruses (CoV) are positive strand RNA viruses that replicate in the cytoplasm of infected cells and produce a nested-set of double-stranded RNA intermediates during viral RNA synthesis [13]. Despite the generation of dsRNA intermediates, CoV infection generally does not induce high levels of IFN production [14]–[18]. The new-emerging and most pathogenic CoV, severe acute respiratory syndrome coronavirus (SARS-CoV) inhibits the induction of IFN-β through blocking translocation of the transcription factor interferon regulatory factor 3 (IRF-3) from the cytoplasm to the nucleus at a later time point in infection [15]. However, activation of innate immunity in specific cell types is likely essential for generating a protective immune response. Studies using knockout mice or siRNA treatment of cell lines indicate that PRR TLR-7 in plasmacytoid dendridric cells [19]; MDA5 in brain macrophages [20], MDA5 and RIG-I in oligodendrocytes [21], and the adapter protein MyD88 are critical for activation of the innate response and protection from lethal coronavirus infection [22]. These studies are consistent with the idea that coronavirus infection induces a type I interferon response in a subset of cells and that the ability to mount an effective innate immune response is essential for clearing the viral infection and generating protective immunity. Furthermore, recent studies indicate that the inefficient activation of the innate immune response may contribute to development of more severe disease [23], [24]. At least two mechanisms have been proposed to explain the low level of type I interferon response to coronavirus infection: the sequestering of viral RNA in double membrane vesicles [25], [26] which prevents or reduces recognition by PRRs; and/or the expression of viral proteins that antagonize the innate response (reviewed in [13]). The most pathogenic CoV, severe acute respiratory syndrome coronavirus (SARS-CoV), which resulted in a 10% mortality rate, encodes at least 6 innate immune antagonists, including nonstructural protein 1 (nsp1) [27], the papain-like protease domain in nsp3 [17], nucleocapsid protein [28], [30], membrane protein [29] and the products of open reading frame 6 (ORF6) and ORF3b [30]. Another important human CoV is NL63, which causes croup in children and is associated with pneumonia in the elderly [31]. HCoV-NL63 also encodes a papain-like protease, termed PLP2, which antagonizes IFN induction [14]. These coronavirus papain-like protease domains (PLPs) are contained within the nonstructural protein 3 (nsp3), which is expressed as part of a replicase polyprotein. The PLPs along with a 3C-like protease (3CLpro) cleave the replicase polyprotein to generate nonstructural proteins (nsp's) that associate with ER membranes to generate convoluted membranes and double membrane vesicles (DMVs), which are the site of viral replication [25], [26]. The CoV PLPs are tethered to the DMVs by a transmembrane domain (Figure 1A). Analysis of enzymatic activity and structural studies revealed that SARS-CoV PLpro and HCoV-NL63 PLP2 function as both proteases and deubiquitinating (DUB) enzymes [14], [32]–[35]. Initially, we speculated that CoV PLPs may act as IFN antagonists via their protease or DUB activities, however we found that both catalytic dependent and catalytic independent mechanisms contribute to PLP-mediated IFN antagonism [14], [17]. Our previous studies indicated that SARS-CoV PLpro inhibits host antiviral innate immune response by inhibiting phosphorylation, dimerization and nuclear translocation of IRF-3, likely by forming a complex with IRF-3 [17]. However, the precise mechanism by which CoV PLPs inhibit IRF-3 activation is still unclear. In this work, we demonstrate that CoV PLPs antagonize IRF-3 signaling by targeting the IRF-3 scaffolding protein STING for inhibition. We also characterize both catalytic-dependent and catalytic-independent roles for PLPs in blocking the activation of IFN response.

## Results

### CoV PLPs antagonize STING-mediated activation of IRF-3

To determine if CoV PLPs are capable of blocking STING-mediated activation of an IRF-3 dependent promoter [36], [37], we assessed the level of IFN stimulated response element reporter (ISRE-Luc) activity in the presence of STING with increasing amounts of coronavirus PLPs. Stimulation of HEK-293T cells with STING alone resulted in greater than 20-fold increase in activity of the ISRE-dependent reporter. Co-expression of STING with wild-type PLP2-TM or PLpro-TM resulted in a dose dependent decrease in ISRE activity indicating that these PLPs can antagonize STING-mediated activation of an IRF-3 dependent promoter (Fig. 1 B and C and supporting information (SI) Fig. S1). To determine if this antagonism is dependent on PLP catalytic activity, cells were co-transfected with plasmid DNA expressing STING and catalytic cysteine mutants of either PLP2-TM or PLpro-TM. Consistent with previous studies, CoV PLP catalytic mutants also act as antagonists, although they are less effective than wild-type PLPs in antagonizing the IFN response [14], [17]. The effect of PLP2-TM on STING-mediated activation was also visualized using confocal microscopy. HEK-293T cells were transfected with STING-HA in the absence or presence of PLP2-TM and the localization of IRF-3 was monitored by immunofluorescence assay. In cells expressing STING-HA, IRF-3 translocates to the nucleus. However, in cells co-expressing PLP2-TM, IRF-3 remains in the cytoplasm (Fig. 1D). In addition, STING-HA and PLP2-TM co-localize in the cytoplasm of transfected cells. These results indicate that CoV PLPs antagonize STING-mediated activation of IRF-3.

### CoV PLPs associate with STING

**Figure 2:**
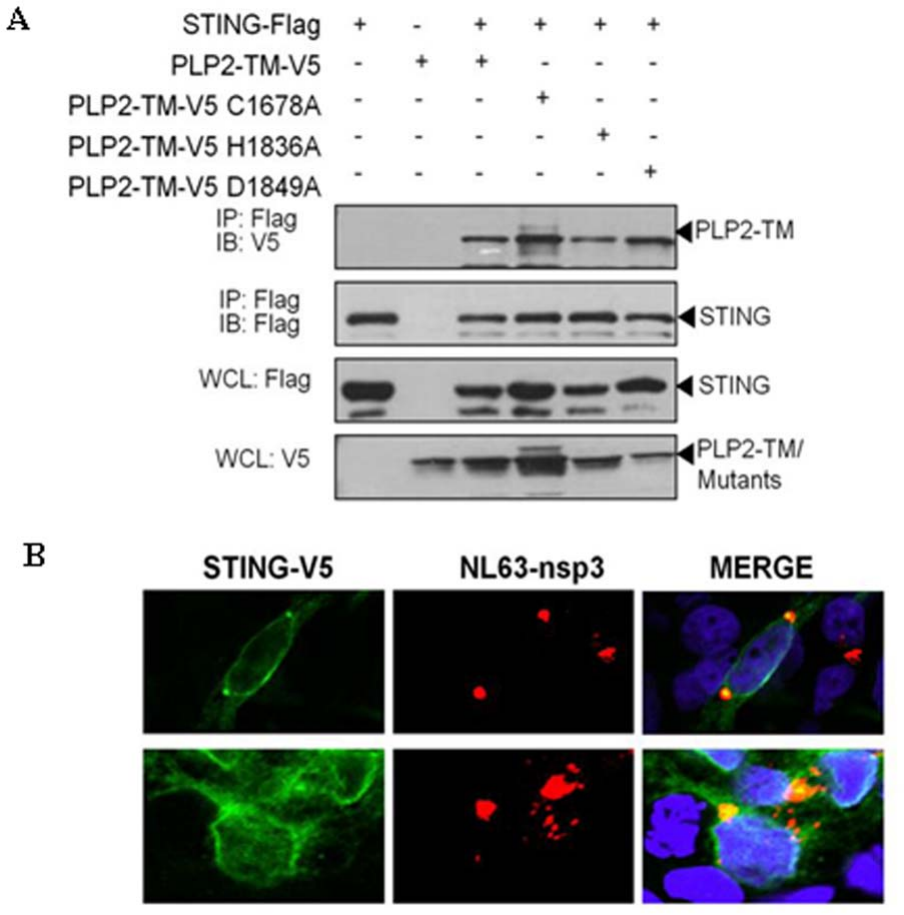
Coronavirus NL63 PLP2-TM associates with STING and nsp3 co-localizes with STING in virus-infected cells. (**A**) HEK293T cells were cotransfected with plasmid DNAs expressing STING-Flag and either wild type or catalytic mutants of NL63-PLP2-TM-V5. Cell lysates were prepared at 28 hrs post-transfection and subjected to immunoprecipitation (IP) with anti-Flag antibody. The products of the immunoprecipitation were separated by SDS-PAGE and subjected to immunoblotting (IB). STING-Flag, PLP2-TM-V5 and the catalytic mutant expression were selectively detected from whole cell lysates (WCL) using anti-Flag and anti-V5 antibodies. (**B**) HEK293-ACE2 cells were transfected with STING-V5 for 4 hours and then infected with HCoV-NL63 for 24 hrs and evaluated for expression of and localization of replicase product nsp3 (anti-nsp3, red) and STING-V5 (anti-V5, green).

One possible mechanism for HCoV PLPs antagonism of STING-mediated activation of IFN is to associate with STING, either directly or as part of a multi-protein complex. Co-immunoprecipitation experiments were performed to determine if CoV PLPs associate with STING. HEK-293T cells were co-transfected with plasmid DNA expressing an epitope tagged version of STING (STING-Flag) in the presence or absence of PLP2-TM and cell lysates were subjected to immunoprecipitation with anti-Flag antibody. The products of the immunoprecipitation were separated by SDS-PAGE and visualized by immunoblotting (Fig. 2A). The results show that both NL63 wild-type and catalytic mutant PLPs are detected in association with STING. Similar co-immunoprecipitation results were obtained using PLpro-TM (Fig. S2) Next, we wanted to determine if STING is sequestered in HCoV-NL63-infected cells. HEK293-ACE2 cells, which express angiotensin-converting enzyme 2 (ACE2), a key receptor for SARS and NL63 coronaviruses, were transfected with STING-V5, infected with HCoV-NL63 and analyzed by confocal microscopy at 24 hrs postinfection. HCoV-NL63 replicase protein nsp3 which contains the PLP2-TM region is detected as punctate, perinuclear staining in virus-infected cells [35], [38]. Interestingly, we detected partial co-localization of STING and nsp3 in virus-infected cells suggesting that STING may be sequestered in the viral replication complex and unable to mediate signaling (Fig. 2B). These results indicate that these CoV PLPs associate with STING either directly or as part of a multi-protein complex.

### STING dimerization is reduced in the presence of CoV PLPs

**Figure 3:**
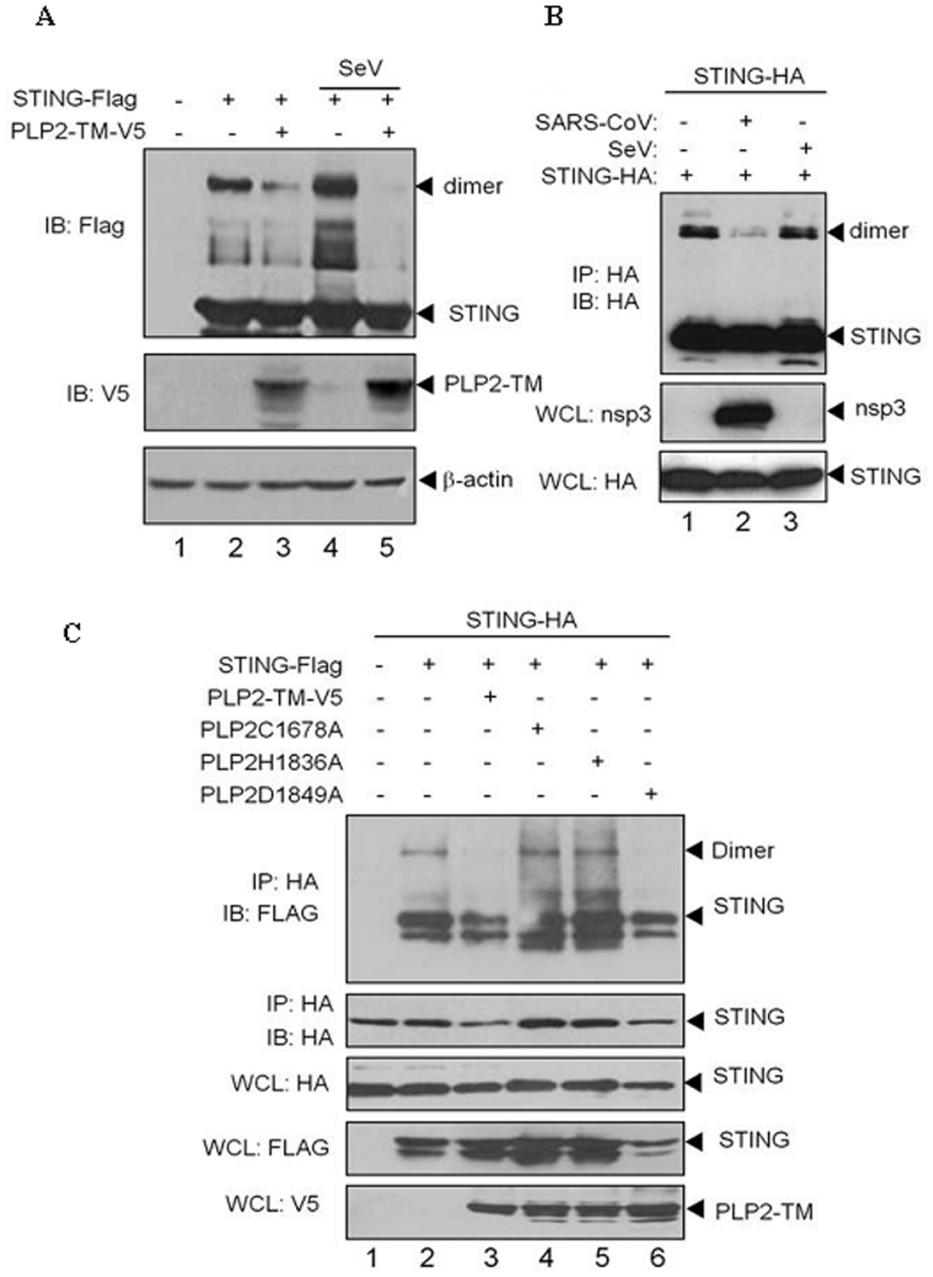
NL63 PLP2-TM interacts with STING and disrupts STING dimers. (**A**) HEK293T cells were co-transfected with plasmid DNA expressing STING-Flag, and/or PLP2-TM and/or infected with Sendai virus (SeV) as indicated above. The cell lysates were separated by SDS-PAGE and subjected to immunoblotting with antibodies as indicated on the left. (**B**) HEK293-ACE2 cells were transfected with plasmid DNA expressing STING-HA and infected with SARS-CoV as indicated and cell lysates were subjected to immunoprecipitation with anti-HA. The immunoprecipitated products were analyzed by SDS-PAGE and immunoblotted to access STING monomers and dimers. Whole cell lysates were immunoblotted to detected SARS-CoV replicase protein nsp3 and STING-HA. (**C**) Cells were co-transfected with STING-HA and STING-Flag with either wild-type or the indicated catalytic mutant of PLP2-TM and lysates were immunoprecipitated (IP) and immunoblotted (IB) to detect expression of each product.

Recent studies indicate that activation of the innate immune response signaling pathway induces dimerization and phosphorylation of STING, which are required for activation of the IFN response [9]. STING dimers can be visualized as a band at 80 kDa when resolved on SDS-PAGE [9]. We hypothesize that PLPs inhibit STING-mediated signaling through the disruption of assembly or stability of STING dimers. To test this hypothesis, cells were co-transfected with plasmid DNA expressing STING-Flag in the presence or absence of PLP2-TM and Sendai virus (SeV), and cell lysates were evaluated for STING dimers by immunoblotting with anti-Flag (Figure 3A). We detected STING dimers in STING-transfected and SeV-infected cells (Fig. 3A, lanes 2 and 4). In contrast, STING dimers were reduced in cells co-expressing PLP2-TM (Fig. 3A, lanes 3 and 5). Similar results were obtained when we evaluated PLpro-TM for disruption of STING dimers (Fig. S3), indicating that these CoV PLPs either prevent assembly or promote dissociation of STING dimers. A similar reduction in STING dimers was seen in cells transfected with STING-HA and infected with SARS-CoV (Fig. 3B, lane 2). In contract, STING dimers were not reduced when infected with SeV (Fig. 3B, lane 3). Collectively, these results indicate that STING dimerization was reduced in the presence of CoV PLPs, and was also substantially reduced in cells infected with SARS-CoV.

To determine if PLP catalytic activity is important for disruption of STING dimers, cells were co-transfected with plasmid DNA expressing STING-HA and STING-Flag and plasmid DNA expressing wt or catalytic mutants of PLP2-TM. Disruption of dimers was assessed by immunoprecipitation and immunoblotting (Fig. 3C). If STING-HA and STING-Flag form heterodimers, then immunoprecipitation with anti-HA and immunoblotting with anti-Flag will allow detection of these heterodimers, as shown in Fig. 3C, lane 2. A reduction in STING dimers was detected in cells expressing wt PLP2-TM and the D1849A mutant which retains DUB activity (lanes 3 and 6), but not in cells expressing the C1678A or H1836A mutants which do not possess DUB activity [33]. These results indicate that PLP2 DUB activity is important for disruption of STING dimers. Similar results were obtained when we evaluated PLpro-TM for disruption of STING dimers (Fig. S3) A previous report indicates that only the dimer form of STING is ubiquitinated [9], which coupled with our observation that PLPs with DUB activity reduce the accumulation of STING dimers, supports a role for DUB activity in negatively regulating STING. Thus, these results are consistent with a role for viral DUB activity in antagonizing either the assembly or stability of STING dimers.

### PLP2-TM disrupts MAVS-STING-IKKε interaction

**Figure 4:**
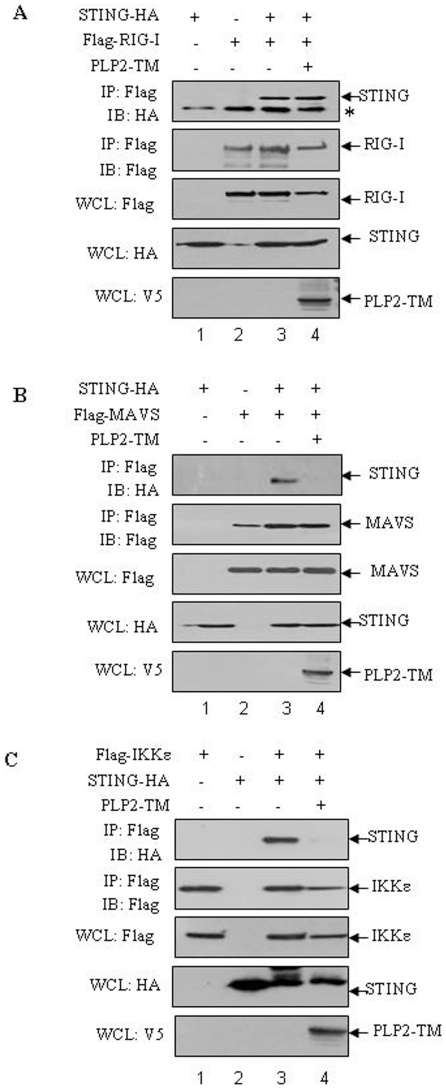
NL63 PLP2-TM disrupts signaling complex formation. HEK293T cells were co-transfected with STING-HA together with either Flag-tagged RIG-I (**A**), Flag-tagged MAVS (**B**) or Flag-tagged IKKε (**C**), and PLP2-TM-V5. At 28 h after transfection, cell lysates were prepared and subjected to immunoprecipitate (IP) and immunoblot (IB) with the indicated antibodies. The asterisk indicates the nonspecific band.

Next, we wanted to determine if PLP2-TM altered the assembly of complexes required for activation of IRF-3 and the IFN response. MAVS, a mitochondrial-associated adaptor protein is a critical player in viral activation of the IFN response. Activation of MAVS mediates the assembly of a multi-protein complex that activates TBK-1/IKKε to phosphorylate IRF-3 [39]. Recent studies revealed that STING associates with MAVS to recruit TBK-1/IKKε and IRF-3 to a complex [8], and that activation of STING is critical for activation of IRF-3 [11], [12]. Therefore, we investigated if PLP2-TM had any effect on assembly of these signaling complexes. HEK-293T cells were co-transfected with plasmid DNAs expressing STING-HA along with Flag-RIG-I, Flag-MAVS or Flag-IKKε in the presence or absence of PLP2-TM. Cell lysates were harvested and evaluated for co-immunoprecipitation of complexes by immunoblotting. We found that expression of PLP2-TM had no effect on co-immunoprecipitation of RIG-I with STING (Fig. 4A), but that co-immunoprecipitation of MAVS and IKKε was disrupted by expression of PLP2-TM (Fig. 4B, lane 4 and Fig. 4C, lane 4). Overall, these results are consistent with an important role for CoV PLPs in blocking activation of IFN by disrupting STING-mediated activation and complex formation.

### PLP2-TM blocks ubiquitination of signaling molecules

**Figure 5:**
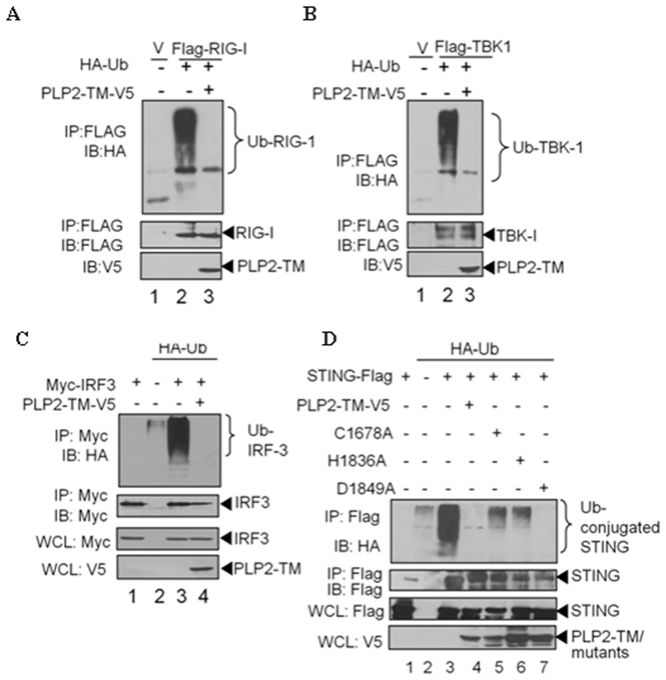
Reduction of ubiquitinated forms of RIG-I, STING, TBK1 and IRF-3 in the presence of NL63 PLP2-TM. HEK293 cells were transfected with Flag-tagged RIG-I(**A**), TBK1(**B**), myc-IRF-3(**C**), or STING-Flag (**D**) together with plasmid DNA expressing HA-tagged Ub in the presence or absence of V5-tagged PLP2-TM-V5. Cells were incubated for 24 hours after transfection and treated with 25 µM MG132 for 4 hours prior to harvesting lysates. Lysates were immunoprecipitated with the indicated antibody and the products were subjected to immunoblotting with anti-HA to evaluate ubiquitinated proteins (upper panels). The whole cell lysates (WCL) were blotted to evaluate expression of each epitope-tagged product (bottom panels).

Modification of signaling molecules by ubiquitination plays a critical role in activation of the IFN response [40]–[42]. Here, we asked if PLP2-TM can recognize and deubiquitinate key complexes in the IFN signaling pathway. HEK-293T cells were transfected with HA-Ub and epitope-tagged versions of either RIG-I, TBK-1, IRF-3 or STING and cell lysates were subjected to immunoprecipitation and immunoblotting to determine the ubiquitination status of the immunoprecipitated proteins (Fig. 5). We found that there was a dramatic reduction in the amount of ubiquitinated RIG-I (A), TBK-1 (B), IRF-3 (C) and STING (D) in cells expressing PLP2-TM. We also investigated the role of the PLP2-TM catalytic activity in mediating deubiquitination. Cells were transfected with HA-Ub and either wild-type or catalytic mutants of PLP2-TM and as expected, we detected a reduction in the level of ubiquitinated STING in the presence of wt and the D1849A mutant of PLP2-TM (Fig. 5D, lanes 4 and 7). Interestingly, expression of the PLP2-TM C1678A and H1836A mutants, which are catalytically inactive [35], still resulted in reduced levels of ubiquitined STING compared to the control (Fig. 5D, lanes 5 and 6). These results suggest that the catalytically inactive mutants of PLP2-TM may block access of STING to the ubiquitination machinery, thereby resulting in reduced levels of Ub-conjugated STING. Thus, the IFN antagonism of the catalytic mutants may be due to physical interaction with STING which blocks access of ubiquitin chains or Ub-ligases or other modifying enzymes that are required for efficient signaling [43], [44]. A previous report indicates that only the dimer form of STING is ubiquitinated [9], which coupled with our observation that PLPs with DUB activity reduce the accumulation of STING dimers, supports a role for DUB activity in negatively regulating STING. Thus, these results are consistent with a role for viral DUB activity in antagonizing either the assembly or stability of STING dimers.

## Discussion

In this study, we investigated the mechanisms of the IFN antagonism imposed by the coronavirus papain-like proteases (CoV PLPs). Previous studies suggested that CoV PLPs block IFN synthesis by inhibiting virus-activated IRF-3 phosphorylation and nuclear translocation, but the underlying mechanism was unclear [14], [17], [32]. Here we show that both SARS-CoV PLpro-TM and HCoV-NL63 PLP2-TM associate with an ER-associated protein, STING and block assembly or stability of STING dimers which are important for downstream signaling and induction of the IFN response. Importantly, we have demonstrated that STING colocalizes with nsp3 (which contains PLPs) in HCoV-NL63 infected cells, and that STING dimerization was substantially reduced in cells infected with SARS-CoV. In addition, we found that ubiquitination of signaling molecules is dramatically reduced in the presence of HCoV-NL63 PLP2-TM, which may contribute to destabilizing the signaling complex. Targeting of the signaling complex by blocking ubiquitination and disruption of STING dimers presents yet another mechanism used by coronaviruses to prevent activation of innate immunity and illustrates how coronavirus PLPs negatively regulate the IFN antiviral immune response in host cells.

### Targeting the stimulator of IFN genes, STING

**Figure 6:**
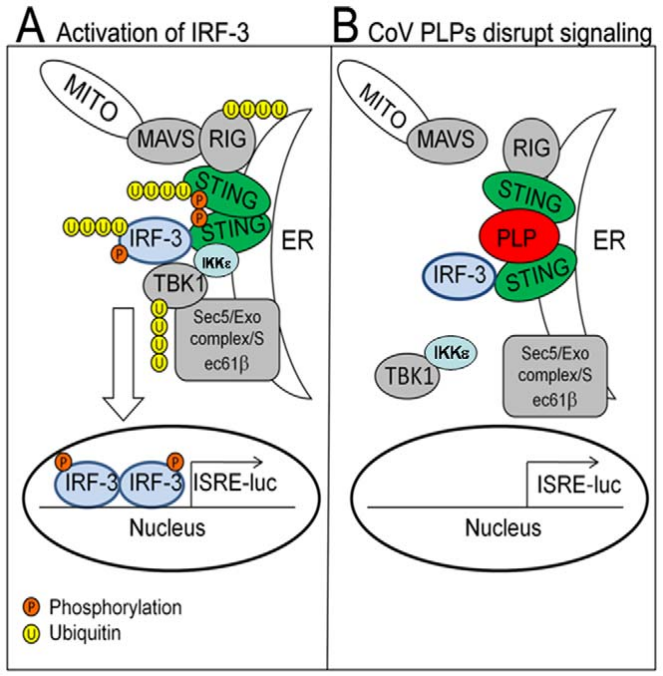
Model depicting the mechanisms used by CoV PLPs to block STING from signaling the activation of the IFN-β induction pathway. (**A**) Activation of sensors such as RIG-I induces interaction with the signaling complex including MAVS, STING, IRF-3 and TBK-1. Activated MAVS interacts with STING, which dimerizes, leading to the activation of IKK complex, TBK1 and IKKε [8]. The activation of this complex leads to the ubiquitination of RIG-I, STING, IRF-3 and TBK1 and the phosphorylatin of STING and IRF-3. Activated the transcription factor IRF-3 translocates to the nucleus inducing production of IFN. (**B**) Coronavirus papain-like protease domains (depicted here as PLP) interact with STING to block signaling by blocking assembly or stability of STING dimers and preventing the ubiquitination of signaling proteins, such as RIG-I, TBK1, and IRF-3.

Recent studies have revealed the arsenal of proteins that viruses use to evade and subvert recognition by pattern-recognition receptors (PRRs) or activation of signaling molecules that are designed to respond to infectious agents [45]. STING is a key scaffolding protein that links the cytosolic viral RNA sensors RIG-I, rather than the MDA5, to the mitochondria protein MAVS [8], [11]. These cytosolic sensors have been shown to be important for recognition of coronavirus RNA in oligodendrocytes [21] and brain macrophages/microglia [20]. Activation of STING, either by expression of N-RIG, the constitutive active caspase recruitment domain of RIG-I or ectopic expression of STING itself, induces the formation of STING dimers, which are modified by phosphorylation and ubiquitylation [9]. The activation of STING facilitates the recruitment of IRF-3 and TBK-1 into a complex where IRF-3 is phosphorylated. Phosphorylated IRF-3 forms dimers and is transported to the nucleus to activate transcription of type I IFN genes. CoV PLPs target STING and prevent this key scaffolding protein from activating IRF-3. Previously, we showed that PLpro blocks NF-κB-dependent promoter activity and that antagonism is abrogated using protease inhibitors [14]. Thus, CoV PLPs interact with key signaling molecules and exploit both catalytic dependent and catalytic-independent mechanisms to block the innate immune response. CoV-PLPs disrupt signal transduction to both IRF-3 and NF-κB, the key transcription factors required for activation of IFN-β (Fig. 6). Interestingly, CoV PLPs seem to exploit both catalytic dependent and independent mechanisms to block STING activity and a catalytic-dependent mechanism to disrupt NF-κB activity.

Previous studies have shown a role for viral proteases in cleavage of key IFN signaling molecules [45]. For example, the NS3/4A protease of hepatitis C virus and GB virus B and the 3ABC precursor of hepatitis A virus cleave MAVS/IPS-1, the mitochondria-associated signaling molecule, which blocks activation of IFN synthesis [46]–[51]. Therefore, it seemed reasonable to hypothesize that CoV PLPs exploited their protease or DUB activity to antagonize the innate immune response. However, we had previously shown that catalytically defective PLP mutants were still capable of inhibiting IRF-3 activation [17]. Furthermore, addition of a protease inhibitor that blocks both protease and DUB activity [52] failed to abrogate the PLP inhibition on activation of IRF-3 dependent promoters [14]. This led us to suspect that CoV PLPs were interacting with a component in the IFN signaling pathway. The fact that CoV PLPs are expressed as a polyprotein that localizes to the ER [25], [26] led us to evaluate ER resident signaling molecules as targets of antagonism. The results presented in this study indicate that CoV PLPs are particularly potent antagonists because they can block: 1) STING dimerization; 2) the MAVS-STING-IKKε interaction required for signaling and 3) the ubiquitination of key signaling molecules such as RIG-I, STING, IRF-3 and TBK-1. Interestingly, catalytically inactive mutants of PLPs can interact, either directly or as part of a signaling complex, with STING and moderately inhibit IRF-3 activation for IFN induction, but wild type PLPs exhibit the most robust inhibition. We found that catalytic activity was important for blocking either the assembly or the stability of STING dimers. In addition, we found that PLP2-TM either actively deubiquitinates or blocks the ubiquitination of the signaling molecules. This is consistent with these reported works that STING dimers were modified by ubiquitin [9]. By associating with STING, either directly or as part of a signaling complex, CoV PLPs can target and either block or deubiquitinate this important signaling molecule. Of course the question still remains if the PLP-STING interaction is direct or indirect, and what region(s) of the PLP are required for this potential interaction. These studies are currently ongoing. Understanding how to negatively regulate STING-mediated innate antiviral signaling by CoV PLPs may lead to the development of novel antiviral therapies and new insights for regulating the IFN response during acute and chronic infections.

### DUBs as negative regulators of IFN activation

One of the striking findings in this report is the similarity in the function of CoV PLPs and cellular DUBs such as the NF-κB responsive gene A20, deubiquitinating enzyme A (DUBA), and the tumor suppressor protein associated with cylindromatosis (CYLD) [53]–[55]. Like CoV PLPs, cellular DUBs were identified as negative regulators of the innate immune response. A20 is considered a “central gatekeeper in inflammation and immunity” [56] because of its ability to interact with and mediate ubiquitin-editing on signaling molecules such as RIP1, TRAF6, RIP2 and NEMO. A20 DUB activity removes K-63 linked polyubiquitin chains from RIP1, TRAF6, RIP2 and NEMO which results in negative regulation of the innate immune response. In addition, A20 has been shown to act by antagonizing interactions between signaling molecules and ubiquitin conjugating enzymes [57]. Interestingly, both A20 and CoV PLPs can act on K-63 linked ubiquitin chains [14], [58]. Further experiments are needed to determine if recognition, processing or sequestering of K-63 linked ubiquitin is important for CoV PLPs negative regulation of the IFN response. Cellular proteins DUBA and CYLD also negatively regulate the innate immune response. Kayagaki and co-workers found that DUBA targets and deubiquitinates TRAF3, a signaling molecule required for activation of IRF-3 [55]. They showed that reducing the expression of DUBA augments the IFN response to poly(I:C) whereas ectopic expression of DUBA blocks the IFN response. CYLD has been shown to deubiquitinate RIG-I to inhibit IFN production [53], [54]. Ectopic expression of CYLD antagonizes the IFN response whereas siRNA-mediated knockdown of CYLD expression allows for a more robust IFN response. It seems that CoV PLPs are usurping the function of cellular DUBs by behaving as negative regulators of the innate immune response through targeting STING for both deubiquitination and dimer disruption.

Another possibility is that CoV PLPs function by sequestering STING as a mechanism of blocking activation of IFN. Both catalytically active and inactive CoV PLPs could interact and sequester STING and thereby prevent activation of IRF-3. Coronavirus PLPs are part of the viral replicase polyprotein that associates with ER membranes to form convoluted membranes and double membrane vesicles (DMVs) which are the sites of viral RNA synthesis [25], [26], [59]. Interestingly, STING also resides in the ER and upon activation assembles with Sec5 into exocyst vesicles [12]. The interaction of STING with PLP2-TM may block the signals required for STING to translocate to exocyst vesicles. Thus, further studies with CoV PLPs may reveal specific targets of STING that modulate this arm of the innate immune response.

In summary, the results of this study indicate that HCoV-NL63 and SARS-CoV PLPs inhibit host IFN-β production by targeting and nullifying STING. Blocking this key scaffolding protein prevents activation of IRF-3 and subsequent transcription of IFN-β. The antagonism functions of CoV PLPs are important negative regulators of the innate immune response and may be important in the virulence and pathogenesis of human coronavirus infection. Further characterization of the PLP-STING interaction may provide new targets for antiviral interventions.

## Materials and Methods

### Cells and virus

HEK293T cells [44] were cultured using Dulbecco's modified Eagle's medium containing 10% (v/v) fetal calf serum, supplemented with penicillin (100 U/ml) and streptomycin (100 µg/ml). HEK293-ACE2 cells, which express angiotensin-converting enzyme 2 (ACE2), a key receptor for SARS and NL63 coronaviruses, were kindly provided by Dr. Kui Li (University of Tennessee Health Science Center, Memphis, USA) and cultured as above with the addition of puromycin (10 µg/ml). HCoV-NL63 was propagated in LLC-MK2 cells [35] which were kindly provided by Lia van der Hoek (University of Amsterdam, The Netherlands) and as previously described [35]. SARS-CoV was propagated in VeroE6 cells as previously described [52]. All work with SARS-CoV was performed in a biosafety level 3 facility using approved protocols. Sendai virus was kindly provided to the Chen lab by Dr. Shaobo Xiao (Huazhong Agricultural University, Wuhan, China) or purchased from Charles River Laboratories.

### Plasmid DNAs

DNA constructs containing wild type and catalytic mutants of NL63 PLP2-TM SARS-CoV PLpro-TM and plasmids of IFN-β-Luc, PRD(III-I)4-Luc, ISRE-Luc and HA-tagged Ub were previously described [14], [17]. Flag-hIPS-1(MAVS), BOS-Myc-hIRF3, Flag-hTBK1, Flag-hRIG-I were kindly provided by Dr. Himanshu Kuma and Shizuo Akira (Immunology Frontier Research Center Osaka University, Osaka, Japan). pcDNA3.1-HA-ERIS (designated here as STING-HA) and pCMV14-Flag-ERIS (designated STING-Flag) were kindly provided by Dr. Zhengfan Jiang (School of Life Sciences, Peking University, Beijing, China).

### Luciferase reporter gene assay

HEK293T cells were transfected with the indicated stimulator plasmid DNA (STING-HA), reporter plasmid DNA [pRL-TK, ISRE-Luc, IFN-β-Luc, or PRD(III-I)4-Luc] and either NL63 PLP2-TM or SARS-CoV PLpro-TM using either Lipofectamine 2000 or Mirus LT1 according to the manufacturer's protocol and incubated for 24 hours. Firefly luciferase and *Renilla* luciferase activities were assayed using the Dual Luciferase Reporter Assay Kit (Promega). Data were shown as mean relative luciferase (firefly luciferase activity divided by Renilla luciferase activity) with standard deviation from a representative experiment carried out in triplicate. For statistical analysis, the data between Vector (300 ng) and PLP (PLpro)-TM with various concentrations were subjected to unpaired, two-tailed Student's t test using the Microsoft SPSS 12.0 software, and P values of <0.05 were considered to indicate statistical significance.

### Immunofluorescence assays

HEK293 or HEK293-ACE2 cells were plated on fibronectin treated glass coverslips in 12-well plates. To evaluate localization of STING and NL63 PLP2-TM, plasmid DNA expressing STING-HA (225 ng per well) was transfected in the presence or absence of 300 ng of PLP2-TM using Mirus LT1 according to the manufacturer's protocol. At 16 hours post transfection, cells were fixed with 3.7% formaldehyde for 10 min at room temperature. Cells were then incubated with 1∶200 dilution of rabbit anti-IRF-3 (Active Motif), mouse anti-HA (Covance), and goat anti-V5 (Genscript) in ADPS (PBS+0.1% Triton-×100+5% fetal calf serum) for 1 hour at room temperature. Cells were washed three times with PBS and incubated with 1∶200 dilution of chicken anti-rabbit Alexa Fluor (AF) 488, donkey anti-mouse AF568, and donkey anti-goat AF647 (Molecular Probes) in ADPS for 1 hour in the dark. Following the incubation, cells were washed three times with PBS, mounted, and imaged with the Zeiss LSM-510 confocal microscope.

To evaluate STING localization in NL63 infected cells, HEK293-ACE2 cells were transfected with 100 ng of a plasmid expressing human STING-V5 for 4 hours. Cells were subsequently infected with 200 µl of HCoV-NL63 (1×10^4^ pfu/ml). At 24 hours post infection, cells were fixed with 3.7% formaldehyde for 10 minutes at room temperature. Cells were then diluted with a 1∶1000 dilution of rabbit anti-nsp3 [35] and 1∶1000 dilution of mouse anti-V5 for 1 hour at room temperature in ADPS. Cells were washed three times with PBS and incubated with a 1∶200 dilution of goat anti-rabbit AF568 and chicken anti-mouse AF488, and 1∶1000 dilution of DAPI for 30 minutes at room temperature in the dark. The cells were then washed three times with PBS, mounted, and imaged with the Zeiss LSM-510 confocal microscope.

### Co-immunoprecipitation (Co-IP) analysis

HEK293T cells were seeded on 100-mm dishes at a density of 1×10^6^ cells/dish. 12 hours later, cells were transiently transfected with a total of 10 µg of empty plasmid or indicated expression plasmids using Lipofectamine 2000 (Invitrogen). At 28 hours after transfection, cells were lysed in buffer containing 0.5% Triton-X-100, 150 mM NaCl, 12.5 mM β-glycerolphosphate, 1.5 mM MgCl2, 2 mM EGTA, 10 mM NaF, 1 mM Na_3_VO_4_, 2 mM DTT plus protease inhibitor cocktail (Sigma). The cell extracts were spun down at 5000× g for 10 minutes at 4°C. The protein concentration of each lysate was determined using the BCA Protein Assay (Bio-Rad) and the concentration was adjusted to 1 µg/µl, with 500 µl of lysate used for each IP. The lysates were precleared by adding 20 µl protein A+G Agarose (Beyotime Institute of Biotechnology, China) and 1 µg of normal IgG and incubating for 2 hours at 4°C, followed by spinning down the beads. The precleared supernatant was incubated with the designated antibody [Anti-Flag (Sigma) or anti-HA (MBL)/anti-Myc (MBL)] with rocking overnight at 4°C. The beads-antibody-antigen complex was spun down and washed 3 times with 1 ml of lysis buffer. The proteins were eluted from the beads in 30 µl of 2× SDS-PAGE sample buffer subjected to boiling for 10 min. The sample was separated by SDS-PAGE and transferred to PVDF membrane for western blotting.

### Assessing ubiquitination of signaling molecules in cultured cells

The effect of HCoV-NL63 PLP2-TM on ubiquitinated proteins in cultured cells was assessed as described previously [53], [55]. Briefly, Flag-tagged RIG-I, TBK1, STING, IRF-3 were co-transfected into HEK293T cells together with pcDNA3.1-HA-Ub, plus wild type or catalytic mutant PLP2-TM DNA using Lipofectamine 2000 according to the manufacturer's instructions. Empty vector pcDNA3.1/V5-HisB was used to standardize the total amount of DNA used for transfection. 24 post-transfection, cells were incubated with 25 µM MG132 for 4 hours, and then lysed in 300 µl of RIPA buffer (50 mM Tris-HCl pH 7.4, 150 mM NaCl, 2 mM EDTA, 1% NP-40, 0.1% SDS) containing protease inhibitor cocktail (1 mM, Roche) and 10 µM NEM, Non-covalently bound proteins were dissociated by boiling in 1% SDS, and samples diluted 1∶10 in lysis buffer (50 mM Tris-HCl pH 7.4, 150 mM NaCl, 2 mM EDTA, 1% NP-40) containing protease inhibitor cocktail and 10 µM NEM. The soluble lysates were then immunoprecipitated with anti-Flag antibody followed by washing with RIPA buffer for three times. Flag-tagged proteins were resolved by SDS-PAGE and sequentially blotted with anti-HA and anti-Flag antibodies. 150 µl of lysate was used for each immunoprecipitation reaction. To confirm the PLP and the catalytic mutant expression level, western blotting with anti-V5 antibody (Invitrogen) was used to detect wild type and catalytic mutant PLP2-TM-V5 protein expression.

### Detection of STING dimers

To assess STING dimers, HEK293T cells were transfected with STING-HA or STING-Flag (0.5 µg per 100 mm dish) and lysates subjected to immunoprecipitation and western blotting as described [9] with the indicated antibodies. To detect STING dimers induced by Sendai Virus (SeV) infection, HEK293T cells were transfected with STING-Flag and then infected with SeV (HAU = 100). 24 hours later, cells were lysed and immunoblotted with anti-Flag antibodies. To assess STING dimers in transfected and SARS-CoV- or Sendai Virus (SeV)- infected cells, HEK293-ACE2 cells were seeded at 10^5^ cells/well in twelve well plates. 24 hours later, cells were transfected with either 0.5 µg of mSTING-HA expressing plasmid DNA or 0.5 µg pcDNA3.1V5HisB vector DNA (Invitrogen). Following six hours of incubation, the cells were infected with either SARS-CoV Urbani (MOI = 0.1) or SeV (HAU = 100). 24 hours later, the cells were lysed in 300 µl of lysis buffer containing 0.5% Triton-X-100, 150 mM NaCl, 12.5 mM β-glycerolphosphate, 1.5 mM MgCl2, 2 mM EGTA, 10 mM NaF, 1 mM Na_3_VO_4_, 2 mM DTT plus protease inhibitor cocktail (Sigma). 150 µl of lysate was used for each immunoprecipitation reaction. After preclearing the lysate with protein G magnetic beads (Millipore), 0.5 µg of rabbit anti-HA antibodies (Invitrogen) was incubated with the lysate overnight at 4°C. 25 µl protein G magnetic beads was added to the immunoprecipitation reactions and incubated for 2 hours at 4°C. Protein G magnetic beads were precipitated and washed 3 times with 1 ml lysis buffer. 80 µl of 2× sample buffer containing 10% glycerol, 5% -mercaptoethanol, 3% SDS, 12.5% upper buffer (0.5 M Trizma base and 0.4% SDS), and 0.01 mg bromophenol blue was added to the beads, and protein-antibody complexes were eluted by incubating at 37°C for 30 min. Samples were separated on SDS-PAGE gel and transferred to a PVDF membrane. Blots were incubated with mouse anti-HA antibody (Sigma) or anti-nsp3 antisera [17] at 0.5 µg/µl and 0.125 µg/µl concentrations, respectively. After washing three times in TBS-T buffer, blots were subsequently incubated with either goat-anti-mouse-HRP or donkey-anti-rabbit-HRP (Southern Biotech). Antibody-antigen reactions were detected using the Western Lighting Plus-ECL chemiluminescence reagents from Perkin Elmer.

## Supporting Information

Figure S1 (**A**) Schematic diagram of SARS-CoV illustrating the processing of replicase polyproteins to generate nonstructural proteins (nsp's). The papain-like protease domains, the catalytic residues, and the transmembrane (TM) domain within nsp3 are indicated. (**B**) Western blot detection of STING-V5 and dose response of PLP2-TM-V5 and PLpro-TM-V5.
(TIF)

Figure S2 SARS-CoV PLpro-TM associates with STING. HEK293T cells were cotransfected with plasmid DNAs expressing STING-Flag and either wild type or catalytic mutants of PLpro-TM-V5. Cell lysates were prepared at 28 hrs post-transfection and subjected to immunoprecipitation (IP) with anti-Flag antibody. The products of the immunoprecipitation were separated by SDS-PAGE and subjected to immunoblotting (IB). STING-Flag, PLpro-TM-V5 and the catalytic mutant expression were selectively detected from whole cell lysates (WCL) using anti-Flag and anti-V5 antibodies.
(TIF)

Figure S3 SARS-CoV PLpro-TM interacts with STING and disrupts STING dimers. HEK293T cells were co-transfected with plasmid DNAs expressing STING-HA, and/or PLpro-TM and/or GFP-V5 as indicated above. At 24 hrs post-transfection, cell lysates were subjected to immunoprecipitation with the indicated antibody and the products were separated by SDS-PAGE and subjected to immunoblotting to detect STING monomer and dimer (upper panel). Whole cell lysates (WCL) were immunoblotted to detected expression of STING-HA, PLpro-TM-V5, and GFP-V5 (lower panel).
(TIF)
