# Supplementary figures and images for: Coronavirus Papain-like Proteases Negatively Regulate Antiviral Innate Immune Response through Disruption of STING-Mediated Signaling
Source: PLoS One. 2012 Feb 1;7(2):e30802. doi: 10.1371/journal.pone.0030802 (PMC3270028; doi:10.1371/journal.pone.0030802)

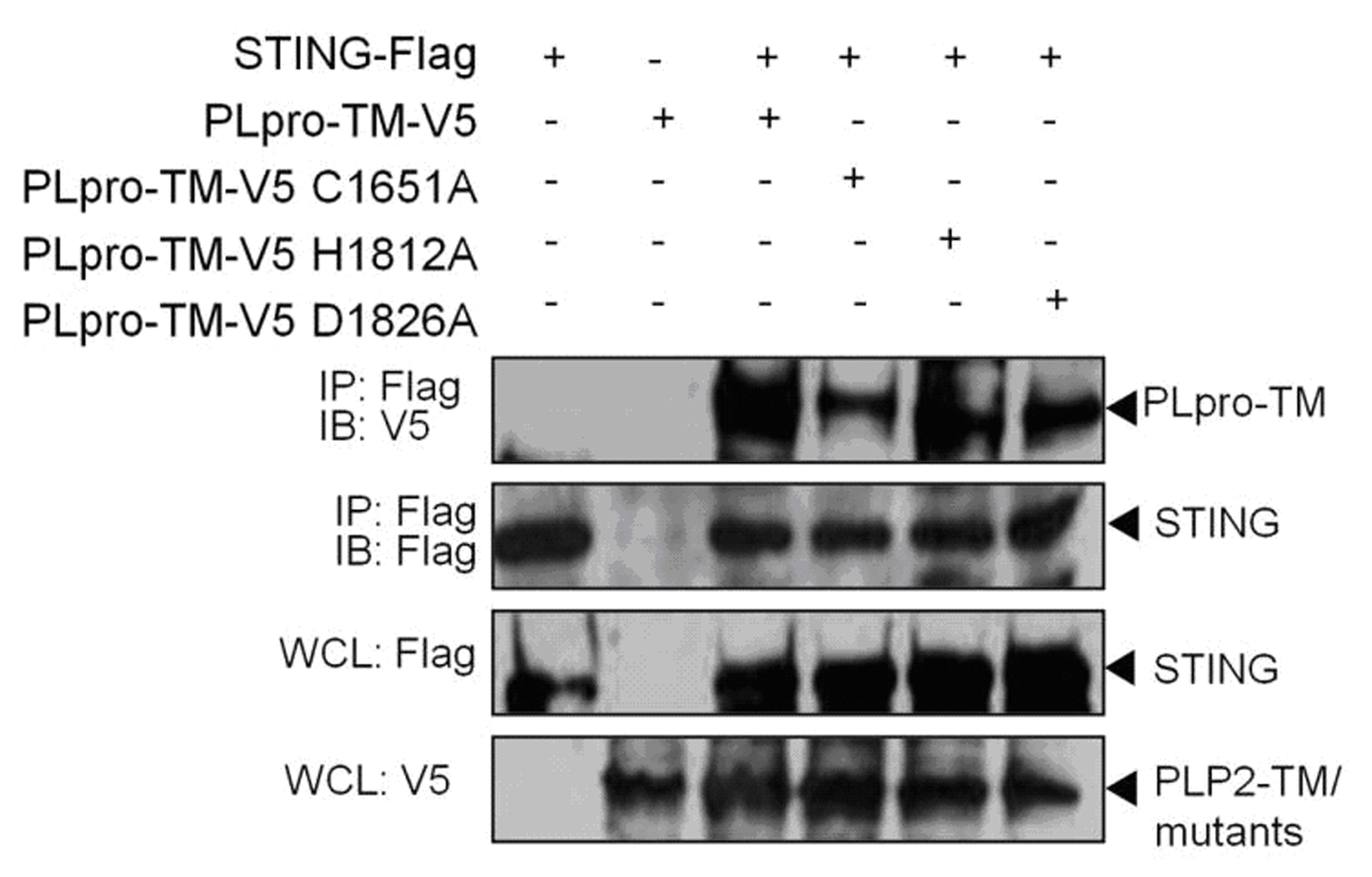

Supplement: Figure S2 — SARS-CoV PLpro-TM associates with STING. HEK293T cells were cotransfected with plasmid DNAs expressing STING-Flag and either wild type or catalytic mutants of PLpro-TM-V5. Cell lysates were prepared at 28 hrs post-transfection and subjected to immunoprecipitation (IP) with anti-Flag antibody. The products of the immunoprecipitation were separated by SDS-PAGE and subjected to immunoblotting (IB). STING-Flag, PLpro-TM-V5 and the catalytic mutant expression were selectively detected from whole cell lysates (WCL) using anti-Flag and anti-V5 antibodies. (TIF) [file pone.0030802.s002.tif]

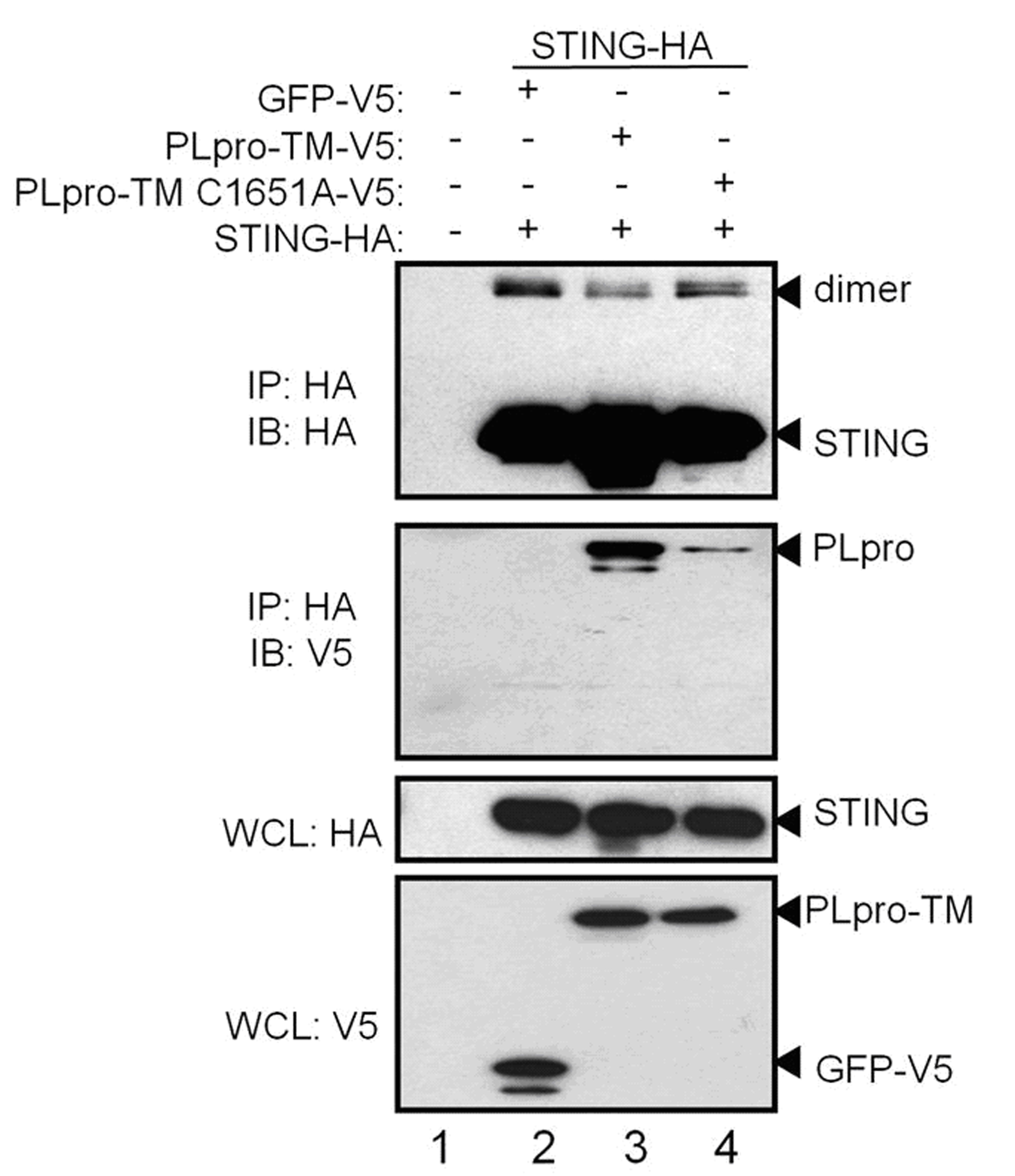

Supplement: Figure S3 — SARS-CoV PLpro-TM interacts with STING and disrupts STING dimers. HEK293T cells were co-transfected with plasmid DNAs expressing STING-HA, and/or PLpro-TM and/or GFP-V5 as indicated above. At 24 hrs post-transfection, cell lysates were subjected to immunoprecipitation with the indicated antibody and the products were separated by SDS-PAGE and subjected to immunoblotting to detect STING monomer and dimer (upper panel). Whole cell lysates (WCL) were immunoblotted to detected expression of STING-HA, PLpro-TM-V5, and GFP-V5 (lower panel). (TIF) [file pone.0030802.s003.tif]
